# Cbfa1 expression in vascular smooth muscle cells may be elevated by increased nitric oxide/iNOS

**DOI:** 10.1590/2175-8239-JBN-2019-0166

**Published:** 2020-05-20

**Authors:** Maria Aparecida da Gloria, Margaret Gori Mouro, Simone Geraldini, Elisa Mieko Suemitsu Higa, Aluizio Barbosa Carvalho

**Affiliations:** 1 Universidade Federal de São Paulo, Departamento de Medicina, Programa de pós-graduação em Nefrologia, São Paulo, SP, Brasil.; 2 Universidade Federal de São Paulo, Departamento de Medicina, Programa de pós-graduação em Medicina Translational, São Paulo, Brasil.

**Keywords:** Core Binding Factors, Kidney diseases, Myocytes, Smooth Muscle, Nitric Oxide Synthase, Fatores de Ligação ao Core, Nefropatias, Miócitos de Músculo Liso, Óxido Nítrico Sintase

## Abstract

**Introduction:**

Vascular calcification is a common complication of chronic kidney disease. Osteoblast differentiation factor (Cbfa1) is present in histologic sections of arteries from patients with end-stage renal disease. Vascular smooth muscle cells (VSMC) can dedifferentiate to osteoblast-like cells, possibly by up-regulation of Cbfa1. There is evidence that the production of nitric oxide (NO) may have an important role in the regulation of osteoblast metabolism. The aim of this study is to evaluate whether increased NO/iNOS expression causes an increase in cbfa1 expression in VSMC.

**Methods:**

VSMC were obtained from renal artery of Wistar male rats, treated for 72 hours with lipopolysaccharide (LPS), ß-glycerophosphate (BGF), a donor of phosphate and aminoguanidine (AG), an inhibitor of iNOS, in the following groups: CTL (control), LPS, BGF, LPS + BGF, and LPS + AG. NO synthesis was determined by chemiluminescence. Cbfa1 and iNOS mRNA expressions were analyzed by RT-PCR, Cbfa1 protein expression by immunohistochemistry and cellular viability by acridine orange.

**Results:**

Cbfa1 and iNOS mRNA expressions were higher in LPS and LPS+ BGF vs CTL (*p* < 0.05), and they were lower in LPS+AG vs LPS (*p* < 0.05). The Cbfa1 in the groups LPS and LPS+BGF also resulted in a higher value compared to CTL (*p* < 0.05), and in LPS+AG it was lower compared to LPS (*p* < 0.05). NO was higher in LPS and LPS+BGF compared to CTL group (*p* < 0.05) and lower in LPS + AG compared to LPS group (*p* < 0.05). Cellular viability showed no statistical difference among groups.

**Conclusion:**

This study showed that increased NO/iNOS expression causes an increase in cbfa1 expression in VSMC.

## RESUMO

**Introdução:**

A calcificação vascular é uma complicação comum da doença renal crônica. O fator de diferenciação osteoblástica (Cbfa1) está presente em cortes histológicos das artérias de pacientes com doença renal em estágio terminal. As células do músculo liso vascular (CMLV) podem desdiferenciar para células do tipo osteoblastos, possivelmente pela regulação positiva da Cbfa1. Há evidências de que a produção de óxido nítrico (NO) pode ter um papel importante na regulação do metabolismo dos osteoblastos. O objetivo deste estudo é avaliar se o aumento da expressão de NO/iNOS causa um aumento na expressão de cbfa1 nas CMLV.

**Métodos:**

As CMLV foram obtidas da artéria renal de ratos machos Wistar, tratados por 72 horas com lipopolissacarídeo (LPS), ß-glicerofosfato (BGF), um doador de fosfato e aminoguanidina (AG), um inibidor da iNOS, nos seguintes grupos: CTL (controle), LPS, BGF, LPS + BGF e LPS + AG. A síntese de NO foi determinada por quimioluminescência. As expressões de mRNA de Cbfa1 e iNOS foram analisadas por RT-PCR, a expressão da proteína Cbfa1 por imunohistoquímica e viabilidade celular por laranja de acridina.

**Resultados:**

As expressões de mRNA de Cbfa1 e iNOS foram maiores em LPS e LPS + BGF v.s. CTL (*p* < 0,05) e menores em LPS + AG v.s. LPS (*p* < 0,05). O Cbfa1 nos grupos LPS e LPS + BGF também resultou em um valor maior em comparação ao CTL (*p* < 0,05), e no LPS + AG foi menor em comparação ao LPS (*p* < 0,05). NO foi maior no LPS e LPS + BGF em comparação ao grupo CTL (*p* < 0,05) e menor no LPS + AG em comparação ao grupo LPS (*p* < 0,05). A viabilidade celular não mostrou diferença estatística entre os grupos.

**Conclusões:**

Este estudo mostrou que o aumento da expressão de NO/iNOS causa um aumento na expressão de cbfa1 nas CMLV.

## INTRODUCTION

The pathogenesis of vascular calcification is not completely understood in clinical practice. Therefore, understanding the regulatory mechanisms that control this calcification is essential for exploring therapeutic targets for potential clinical applications.

Vascular smooth muscle cells (VSMCs) are the cellular components of the normal blood vessel wall that provides structural integrity and regulates the diameter of vessels by contracting and relaxing dynamically in response to vasoactive stimuli^1^.

Vascular calcification is an actively regulated process similar to osteogenesis, in which bone-associated proteins may be involved. The osteoblast differentiation factor (Cbfa1) is a critical factor for osteoblasts, and the expression of bone matrix proteins is thought to be the switch that turns the mesenchymal cell into an osteoblast-like cell and therefore leads to VSMC calcification^2^^,^ 3.

Studies have demonstrated that Cbfa1 controls the expression of osteopontin, type I collagen, and osteocalcin in osteoblasts and it has been associated with enhanced calcification seen in cultured smooth muscle cells^4^. These cells mineralize in the presence of β-glycerophosphate (BGF), a phosphate donor, with upregulation of Cbfa1^5^.

Abnormalities in mineral metabolism, in particular hyperphosphatemia, frequently observed in chronic renal disease, have emerged as a key regulator of vascular calcification and been considered risk factors for cardiovascular mortality in this population^6^^,^ 7. NO is a cell-signaling molecule with multiple roles, including the regulation of vascular tone and neurotransmission^8^^,^ 9. NO is produced by most cells of the body and its substrate is the L-arginine, which under the effect of nitric oxide synthase (NOS) generates L-citrulline and NO^10^. The NO synthesized by endothelial cells is crucial in maintaining vascular health and preventing the development of vascular diseases^11^. NOS presents two isoforms: the constitutive (cNOS) and the inducible (iNOS); the latter can be induced by agents as lipopolysaccharide (LPS) from several bacteria and inhibited by aminoguanidine (AG)^12^. NOS expression has been detected *in vivo* and *in vitro* in osteoblastic cell lineage^13^ and there is evidence that the production of NO may have an important role in the regulation of osteoblast metabolism^14^.

Studies about the effect of NO on osteoblastic function are unclear. Some researchers have shown that NO donors increase cGMP production and alkaline phosphatase activity and induce bone nodule formation *in vitro* 15 ^,^ 16. Therefore, the aim of the present study was to evaluate whether increased NO production by iNOS has an effect on Cbfa1 expression in VSMC from rat renal artery.

## METHODS

This study was approved by the Institutional Animal Ethics Committee.

### VSMC CULTURE

Wistar male rats weighing 250 to 300 g were anesthetized with a mixture of ketamine (80 mg/kg) and xylazine (10 mg/kg) by intraperitoneal injection. Kidneys were isolated through an abdominal incision and the renal arteries were removed and washed in cold phosphate-buffered saline (PBS). The “explant” technique for smooth muscle cell primary culture was used. Cells were grown in 25 cm^2^ culture flasks, in Dulbecco’s modified Eagle’s medium (DMEM) with NaHCO_3_ (2 g/L), Hepes (2.6 g/L), and penicillin (10.000 U/L) supplemented with 20% fetal bovine serum (FBS) and incubated in a humidified atmosphere with 5% CO_2_ at 37◦C. After the first passage, DMEM with 10% FBS was used.

Cells were used in the experiments between the third and tenth passages and were treated during 72 hours^17^ according to the following experimental groups: CTL- control (DMEM 10%); LPS (DMEM 10% + LPS 100 µg/mL); BGF (DMEM 10% + BGF12 mM); LPS+ BGF (DMEM 10% + LPS+BGF); and LPS+ AG (DMEM 10% + LPS+ AG 30 mM).

After 72 hours, cell culture media were collected and stored at -20 °C for nitric oxide determination. Cells were lysed with 2% sodium dodecyl sulfate (SDS) for protein determination.

### DOSE-RESPONSE CURVE

A distinct group of cells was treated for 72 hours with LPS (50, 100, 200, or 300 µg/mL). The dose response curve of NOS was obtained by measuring NO in culture media and iNOS and Cbfa1 mRNA expressions.

### NO DETERMINATION

NO was determined in the cell culture media by the chemiluminescence method^17^. We used the Model 280 Nitric Oxide Analyzer (NOATM) from Sievers Instruments, Inc. (Boulder, CO, USA), a high-sensitive detector for measuring nitric oxide, based on a gas-phase chemiluminescent reaction between nitric oxide and ozone: $\mathrm{NO}+O_{3}\;\to \mathrm{NO}-2+O_{2}\mathrm{NO}-2\;\to {\mathrm{NO}}_{2}+\mathrm{hv}$. The emission from electrically excited nitrogen dioxide is in the red and near-infrared region of the spectrum, and it is detected by a thermoelectrically cooled red-sensitive photomultiplier tube. The sensitivity for measurement of NO and its reaction products in liquid samples is ~ 1 picomole.

### CELLULAR VIABILITY

Cellular viability was assessed by the acridine orange fluorescent dye and ethidium bromide. After cells were trypsinized, a 10 µL sample of cell suspension was incubated with 0.3 µL acridine orange/ethidium bromide solution (100 µL/mL of each dye). Acridine orange is cell permeable and binds to either the double-stranded DNA emitting a green fluorescence (excitation 502 nm and emission 525 nm) or single-stranded RNA emitting a reddish-orange fluorescence (excitation 460 nm and emission 650 nm). In contrast, ethidium bromide binds only to the double stranded DNA and also emits a red fluorescence (excitation 510 nm and emission 595 nm); however, the cell is not permeable to the dye when the plasma membrane is intact, and therefore, only necrotic cells will take up this dye (with the red fluorescence quashing the green/orange of acridine orange). Cell suspensions were observed under a fluorescent microscope at 40X magnification, and 200 cells from a number of microscopic fields were counted. Cells emitting a green fluorescence were considered viable, while those emitting a red fluorescence were considered non-viable. Viable cells were reported as percentage of the total counted cells.

### REVERSE TRANSCRIPTION-POLYMERASE CHAIN REACTION (RT-PCR) OF INOS AND CBFA1

Total RNA was extracted from cultured cells by the TRIZOL^®^ method. The resulting RNA was used to generate the cDNA fragment corresponding to the iNOS by RT-PCR amplification using the primers for iNOS (464 bp): 5´-CCG GAT CCT CTT TGC TAC TGA GAC AGG-3´and 5´-CCG AAT TCG GGA TCT GAA TGC AAT GTT-3´. The cycling parameters were hot-started at 95 ºC for 3 minutes, followed by 40 cycles at 95 ºC for 30 seconds, 55 ºC for 30 seconds, 72 ºC for 30 seconds, 72 ºC for 7 minutes, and 4 ºC. The primers for Cbfa-1(111 bp): 5´-CCT CAC TGA GAG CCG CTT CT- 3´ and 5´-GTA GTG AGT GGT GGC GGA CAT-3´cycling parameters were hot-started at 95 ºC for 3 minutes, followed by 40 cycles at 95 ºC for 45 seconds, 56 ºC for 45 seconds, 72 ºC for 45 seconds, 72 ºC for 10 minutes, and 4 ºC. The RT-PCR products were resolved on a 3% agarose gel and visualized by EB staining. Finally, a figure was obtained by Kodak Electrophoresis Documentation and Analysis System (EDAS), model DC120, USA, using an ethidium bromide filter, coupled with a UV transluminator. The band density was analyzed by scanning densitometry using image Quant 4.0 software (Storm) (Molecular Dynamics, Sunnyvale, CA, USA). Semi-quantitative RNA estimation was carried out with β-actin (191bp) as control. The results are reported as the ratio of arbitrary units of the band densities.

### IMMUNOHISTOCHEMISTRY - CBFA1

A cell suspension (3x10^5^ cells/mL) was used to make thin-layer preparations with the Cytospin cytocentrifuge (Shandon, Pittsburgh, PA USA). Preparations were fixed in acetone and washed in PBS. Cells were incubated in a dark-humid chamber at room temperature for 2 hours with goat polyclonal antibody against Cbfa1 (Santa Cruz Biotechnology, California, USA, 1/100 dilution). After washings in PBS, cells were treated with the secondary antibody and the complex streptavidin-biotin-peroxidase according to the manufacturers of the LSAB (DAKO A/S, Glostrup, Denmark). Hematoxylin was used to counterstain the nuclei. Immunoreactive cells showed dark brown staining cytoplasm. Slides were analyzed by conventional light microscopy (Leica DM1000, Switzerland). Quantification was performed by an automated quantification program microsystem (Leica LAS V3.8, camera DFC310 FX).

### STATISTICAL ANALYSIS

Results were reported as mean ± standard error of mean (SEM). To compare NO synthesis and immunocytochemistry between the groups we used the one-way analysis of variance (ANOVA). The RT-PCR was quantified by densitometry, and the difference in the Cbfa1/β-actin and iNOS/ β-actin ratios between the groups was compared by ANOVA with Tukey’s post-hoc test. Statistical significance was defined as *p* < 0.05.

## RESULTS

All groups presented similar cellular viability to CTL (96.3 ± 2.1%) (*p* > 0.05): control LPS (96.9 ± 2.5%), LPS + AG (93.5 ± 5.1%), LPS + BGF (95.3 ± 2.6%), and BGF (96.2 ± 3.7%).

The dose-response curve of LPS on the NO synthesis is shown in Table 1. NO synthesis was higher in all groups compared to LPS 50 (*p* < 0.05).

**Table 1:** Dose-response curve of NO synthesis (nmol/mg protein) in VSMC exposed to LPS (50, 100, 200, or 300 µg/mL) for 72 hours

Group	LPS	LPS	LPS	LPS
	50 µg/mL	100 µg/mL	200 µg/mL	300 µg/mL
	51.8 ± 3.8	104.5 ± 22.0^a^	123.9 ± 12.3^a^	197.9 ± 44.1^a^

Mean ± SEM, one-way analysis of variance (ANOVA);

a *p* < 0.05 vs LPS 50 µg/mL

**Figure 1:**
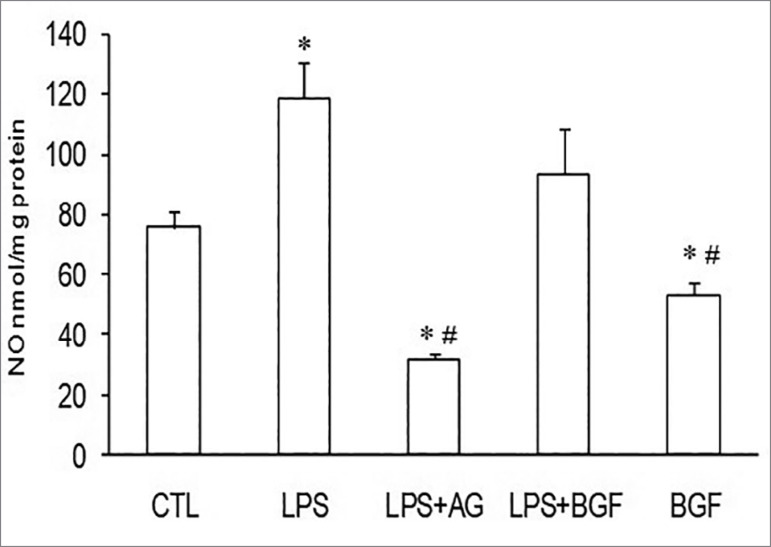
NO synthesis in cultured VSMC treated with LPS (100 μg/mL), LPS+AG (30 mM), BGF (12 mM), or LPS + BGF (12mM), analyzed by the chemiluminescence method. Mean ± SEM (N=5 for all groups). *p < 0.05 vs. control (CTL); #*P* < 0.05 vs. LPS.

Figure 1 shows the results of NO synthesis (nmol/mg protein) by VSMC in the groups: CTL, LPS, LPS + AG, LPS + BGF, and BGF. Comparing to CTL group, NO synthesis was increased in the LPS (118.9 ± 11.6 vs 75.6 ± 5.1; *p* < 0.05) and decreased in the LPS+AG and BGF groups (31.8 ± 1.5 and 53.0 ± 3.9, respectively; *p* < 0.05). Compared to LPS, NO synthesis was decreased in LPS + AG (31.8 ± 1.5; *p* < 0.05) and in the BGF (53.03 ± 3.9; *p* < 0.05). NO synthesis in the LPS+BGF group (93.4 ± 15.7) was increased when compared to the control group and was decreased when compared to the LPS group, although no statistical significance was found.

**Figure 2:**
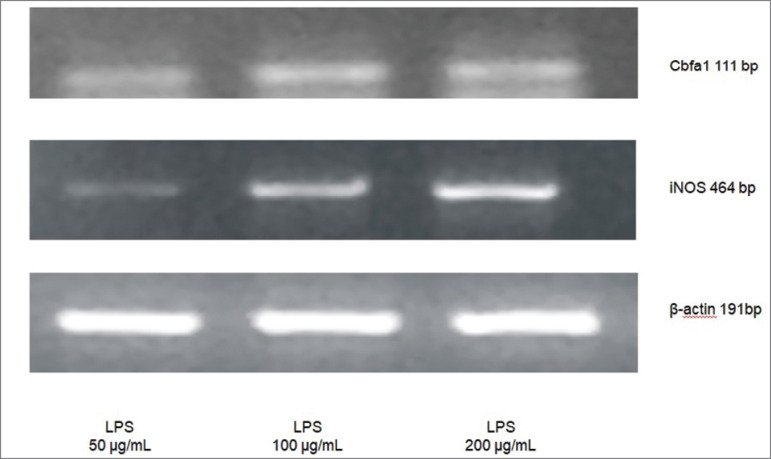
Dose-response of LPS (50, 100, or 200 μg/mL) on Cbfa1 or iNOS mRNA expression in VSMC. Representative RT-PCR amplification products using the primers for Cbfa1, iNOS, or β-actin.

Figure 2 shows the representative gel image of LPS dose response curve on Cbfa1 and iNOS mRNA expressions at 50, 100, or 200 µg/mL. Densitometric analysis of Cbfa1/β-actin showed lower values with LPS 50 (0.20 ± 0.10) compared to LPS 100 (0.36 ± 0.06) or LPS 200 (0.39 ± 0.11) (*p* < 0.05), and iNOS/β-actin showed lower values with LPS 50 (0.35 ± 0.05) compared to LPS 100 (0.63 ± 0.12) or LPS 200 (0.95 ± 0.19) (*p* < 0.05).

**Figure 3:**
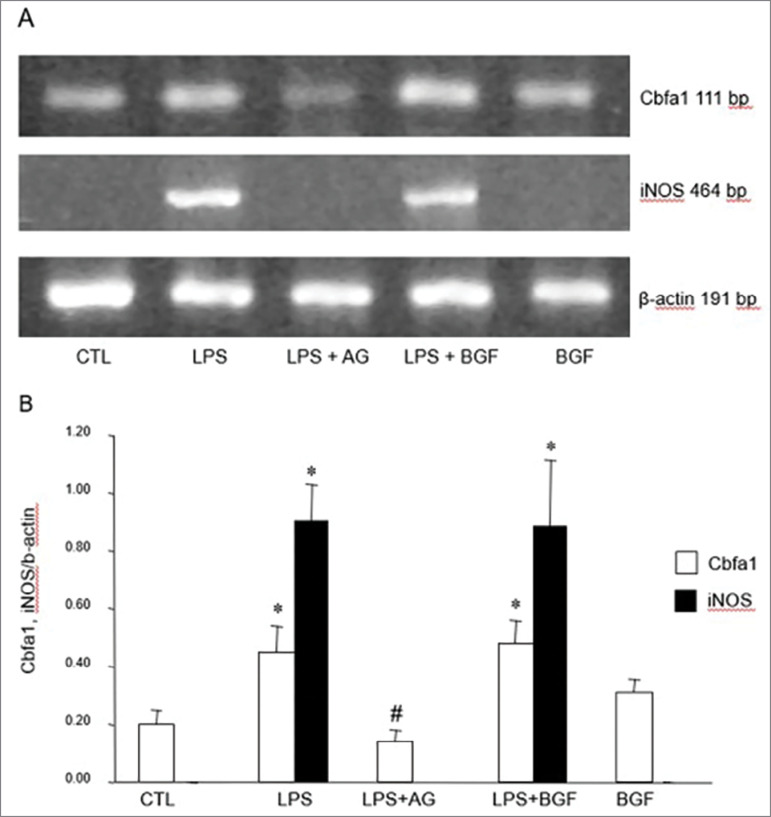
Effects of LPS (100 µg/mL), LPS+AG (30 mM), LPS + BGF (12 mM), or BGF (12 mM) in Cbfa1 and iNOS expression. (A) Representative RT-PCR amplification products using the primers to Cbfa1, iNOS isoform, or β-actin. (B) Densitometric analysis of Cbfa1/β- actin or iNOS/β-actin. *p < 0.05 vs. CTL, #*p* < 0.05 vs. LPS.

Figure 3 shows the representative gel image (A) and densitometric quantification (B) of Cbfa1 or iNOS mRNA in CTL, LPS, LPS + AG, LPS + BGF, or BGF groups. In cells treated with LPS or LPS + BGF, the expression of iNOS mRNA was detected (0.90 ± 0.13 and 0.89 ± 0.23, respectively), while in CTL and in the groups treated with LPS + AG or BGF there was no expression of iNOS mRNA. There was an overexpression of Cbfa1 mRNA in rVSMC treated with LPS (0.45 ± 0.09) or LPS + BGF (0.48 ± 0.07) when compared to control (0.20 ± 0.04; *p* < 0.05, for both), while in those treated with LPS+AG (0.14 ± 0.04) the Cbfa1 mRNA expression was lower when compared to LPS group (*p* < 0.05).

**Figure 4:**
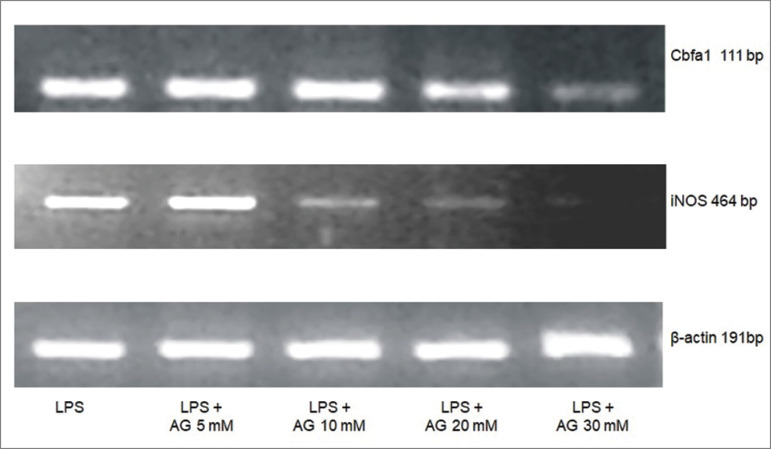
Dose-response of AG (5, 10, 20, and 30 mM) on Cbfa-1 or iNOS mRNA expressions after 72 hours of treatment with LPS (100 µg/mL) in cultured VSMC. Representative RT-PCR amplification products using the primers for Cbfa1, iNOS isoform, or β-actin.

Figure 4 shows the representative gel image of AG dose response on Cbfa1 or iNOS mRNA, in cells treated with LPS 100 µg/mL. As for Cbfa1 mRNA expression, no difference was observed at doses of 5, 10, or 20 mM of AG when compared to LPS alone. Cbfa1 mRNA expression at the dose of 30 mM of AG (0.11 ± 0.04) was lower as compared to LPS alone (0.56 ± 0.09) (*p* < 0.05). iNOS mRNA expression was also lower at 30 mM of AG (0.47 ± 0.06) when compared LPS alone (*p* < 0.05).

**Figure 5:**
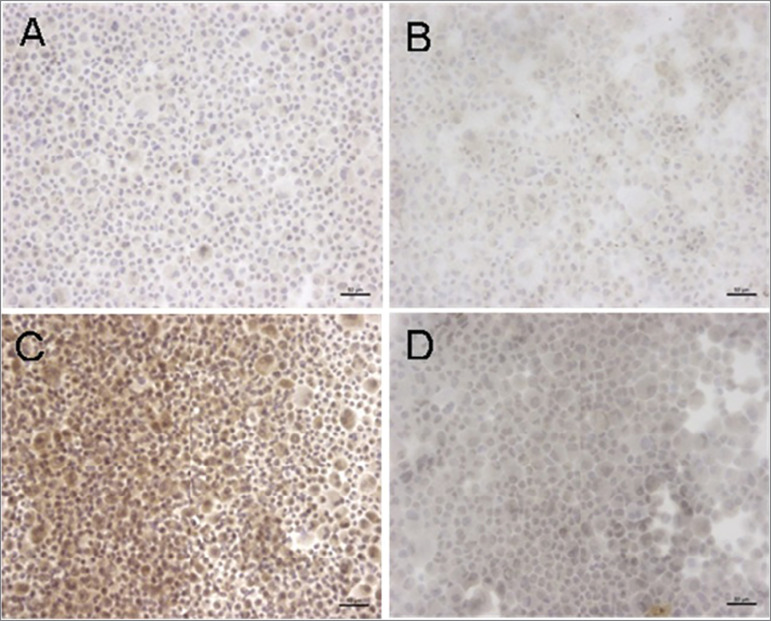
Cbfa1 immunocytochemistry. **A**- CTL, **B**- BGF (12 mM), **C**- LPS (100 µg/mL), **D**- LPS (100 µg/mL) + AG (30mM).

Figure 5 shows the images of Cbfa1 protein expression in the groups CTL (A); BGF (B); LPS (C), and LPS + AG (D).

**Figure 6:**
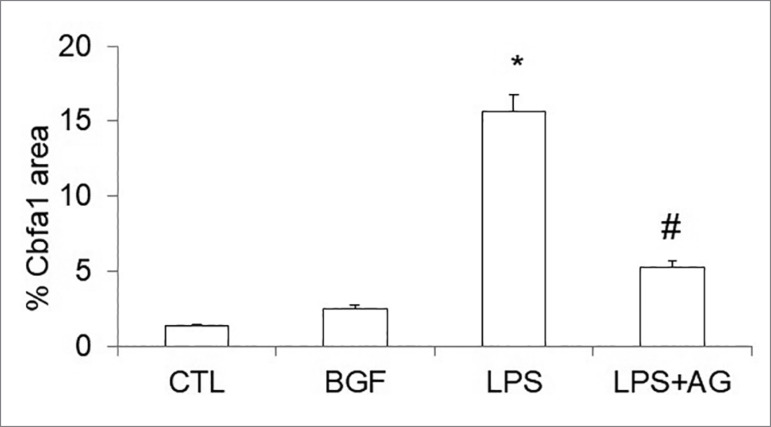
Quantification of immunocytochemistry. Area stained Cbfa1. * *p* < 0.05 vs CTL; # *p* < 0.05 vs LPS.

Figure 6 shows the percentage of Cbfa1 stained area. Cbfa1 expression was increased in LPS group when compared to CTL group (15.64 ± 1.16 vs 1.36 ± 0.12; *p* < 0.05), and in the LPS + AG group it was decreased when compared to LPS (5.25 ± 0.44 vs 15.64 ± 1.16; *p* < 0.05).

## DISCUSSION

In the present study, mRNA and protein expressions of Cbfa1, iNOS, and NO synthesis in cultured VSMC were evaluated. We found that Cbfa1 expression increased when NO production increased due to increased iNOS expression.

The mRNA expression of Cbfa1 and iNOS and NO levels were higher in LPS and LPS + BGF, and lower when AG was added. The Cbfa1 analyzed by immunohistochemistry in the groups LPS or LPS+BGF were higher when compared to CTL.

According to our NO dose response curve in the VSMC treated with LPS (Table 1), a progressive increase of NO synthesis from LPS 50 to 300 µg/mL was observed. The dose of 100 µg/mL was used since it has been previously demonstrated that this dosage was able to induce iNOS expression and increase NO synthesis^17^.

NO synthesis was increased in the presence of LPS and decreased when AG was added; however, in the presence of BGF, we cannot explain why a decrease in NO production occurred. Besides, up to now, there are no data in the literature concerning this issue (Figure 1).

LPS is usually able to induce iNOS expression and increase NO production within 24 hours. Nevertheless, according to Jono et al.^2^, 72 hours is an ideal time for increasing Cbfa1 expression after addition of BGF in cultured VSMC, maintaining cell viability. Also, the metabolites of NO, nitrites and nitrates, are stable in 72 hours.

In the dose response curve of mRNA Cbfa1 and iNOS, there was a higher value at doses of 100 and 200 µg/mL after 72 hours, as shown in Figure 2.

In the Figures 3A and 3B, we observed that iNOS mRNA was overexpressed when cells were treated by LPS and it was inhibited by AG. Regarding Cbfa1 expression, our results showed a similar behavior, i.e., an overexpression of Cbfa1 in cells treated with LPS and an inhibition of the gene when AG was added in the culture. Whether there is or not a direct mechanism leading to increased Cbfa1 expression through of iNOS needs further discussion.

Experiments performed in our laboratory with VSMC treated only with AG showed no difference in Cbfa-1 expression. The dose of 30 mM of AG was chosen because it was the optimum dose for inhibiting iNOS and Cbfa1 after 72 hours (Figure 4). Although some investigations used the dose of AG around 100 µM to inhibit the NOS expression, protocols established by other studies ranged from 10 to 100 mM^18^^,^ 19.

Some studies have investigated the relationship of NOS isoforms with Cbfa1 in osteoblast cells^20^^,^ 21. It is known that both VSMC and osteoblast cells come from the same mesenchymal origin^22^^,^ 23. Furthermore, in some disease states, VSMC may dedifferentiate to osteoblast-like cells, a mechanism mediated by Cbfa1^24^. Jono et al.^1^ first demonstrated that VSMC mineralize in the presence of BGF at 12 mM, with upregulation of Cbfa1^1^. On the other hand, Moe et al.^4^ demonstrated that in bovine VSMC incubated with uremic serum from patients on hemodialysis, there was an upregulation of Cbfa1 through a non-phosphorus-mediated mechanism, suggesting that the etiology of vascular calcification in dialysis patients is multifactorial. To test the effects of phosphorus on Cbfa1 expression, we added BGF in the cultured VSMC. Differently from Jono’s study, we could not observe an overexpression of Cbfa1 after increasing the phosphate concentration. There was no overexpression of Cbfa1 mRNA in cells after addition of BGF and we could not observe an overexpression of this gene after treatment with LPS+BGF; this overexpression was similar to that of LPS alone, confirming the absence of BGF effects on Cbfa1.

Cbfa1 protein expression by immunohistochemistry was higher in the presence of LPS compared to the CTL and BGF groups; in the presence of AG, it was lower compared to LPS group as shown in Figures 5 and 6.

Our results are in agreement with those of Moe et al., showing that Cbfa1 expression by VSMC can be induced by a non-phosphorus-mediated mechanism, among others^4^, such as reactive oxygen species.

Although oxidative stress was not evaluated in this study, it should be remembered that reactive nitrogen species act in conjunction with reactive oxygen species, damaging cells and causing nitrosative stress^8^. These two species are therefore often referred to collectively and would have to be better evaluated in future studies.

Although there was an increase in Cbfa1 expression following increased NO/iNOS expression, it cannot be said it was due to either a direct or indirect path. To support this hypothesis, further investigations about mechanisms will be needed for a better understanding of the complex relationship between NO system and the Cbfa1 gene expression.

## CONCLUSION

In conclusion, this study showed that increased NO/iNOS expression causes an increase in cbfa1 expression in cultured VSMC, indicating that increased NO production may participate on Cbfa1 expression.
